# High clonality of *Mycobacterium avium* subsp. *paratuberculosis* field isolates from red deer revealed by two different methodological approaches of comparative genomic analysis

**DOI:** 10.3389/fvets.2024.1301667

**Published:** 2024-02-06

**Authors:** Silvia Turco, Simone Russo, Daniele Pietrucci, Anita Filippi, Marco Milanesi, Camilla Luzzago, Chiara Garbarino, Giorgia Palladini, Giovanni Chillemi, Matteo Ricchi

**Affiliations:** ^1^Dipartimento di Scienze Agrarie e Forestali (DAFNE), Università degli Studi della Tuscia, Viterbo, Italy; ^2^National Reference Centre and WOAH Reference Laboratory for Paratuberculosis, Istituto Zooprofilattico Sperimentale della Lombardia e dell'Emilia Romagna “Bruno Ubertini”, Piacenza, Italy; ^3^Dipartimento per l'Innovazione nei Sistemi Biologici, Agroalimentari e Forestali (DIBAF), Università degli Studi della Tuscia, Viterbo, Italy; ^4^Department of Veterinary Medicine and Animal Sciences, Coordinated Research Centre "EpiSoMI", University of Milan, Lodi, Italy; ^5^Institute of Translational Pharmacology, National Research Council, CNR, Rome, Italy

**Keywords:** *Mycobacterium avium* subsp. *paratuberculosis*, red deer, wild animals, Italy, whole genome sequencing, epidemiology

## Abstract

*Mycobacterium avium* subsp. *paratuberculosis* (MAP) is the aetiological agent of paratuberculosis (Johne’s disease) in both domestic and wild ruminants. In the present study, using a whole-genome sequence (WGS) approach, we investigated the genetic diversity of 15 *Mycobacterium avium* field strains isolated in the last 10 years from red deer inhabiting the Stelvio National Park and affected by paratuberculosis. Combining *de novo* assembly and a reference-based method, followed by a pangenome analysis, we highlight a very close relationship among 13 MAP field isolates, suggesting that a single infecting event occurred in this population. Moreover, two isolates have been classified as *Mycobacterium avium* subsp. *hominissuis*, distinct from the other MAPs under comparison but close to each other. This is the first time that this subspecies has been found in Italy in samples without evident epidemiological correlations, having been isolated in two different locations of the Stelvio National Park and in different years. Our study highlights the importance of a multidisciplinary approach incorporating molecular epidemiology and ecology into traditional infectious disease knowledge in order to investigate the nature of infectious disease in wildlife populations.

## 1 Introduction

*Mycobacterium avium* subsp. *paratuberculosis* (MAP), a member of the *Mycobacterium avium* complex, is an important pathogen responsible for a chronic granulomatous enteritis known as paratuberculosis or Johne’s disease (1). The genome of MAP shares very high homology with that of the other *Mycobacterium avium* subspecies, but, to date, some sequences, such as the F57 sequence, have been found only in MAP isolates, and it is considered one of the more specific targets for the diagnosis of paratuberculosis by PCR (2). MAP has been isolated from both ruminant and non-ruminant hosts, and, among wild animals, the red deer (*Cervus elaphus*) is one of the more affected animal by this disease (3). For this reason, according to the new Animal Law Regulation 429/2016, by being a natural MAP reservoir, the red deer is a potential epidemiological risk for MAP transmission in the surrounding area. From a clinical perspective, the manifestations of paratuberculosis in red deer closely resemble those observed in cows: persistent diarrhoea, weight loss, and deteriorating body condition as the disease advances (4). For a genetic perspective, three major MAP strain types were initially identified according to the host species and based on restriction endonuclease analysis, DNA hybridisation, and pulsed-field gel electrophoresis (PFGE): (*i*) Type I or S type for sheep; (*ii*) Type II or C type for cattle; and (*iii*) Type III or “intermediate” between Type I and Type II (5–8). A fourth strain type named “Bison” or Type B was later isolated from bison (*Bison bison*) in Montana, United States and distinct from an Indian Bison Type (9, 10).

Numerous studies have already been addressed at catching the diversity among MAP field isolates from red deer in European Countries. In 2009, Stevenson et al. characterised 164 MAP strains isolated from 19 diverse host species through restriction fragment length polymorphism (RFLP), PFGE, amplified fragment length polymorphism (AFLP), and mycobacterial interspersed repeat unit-variable number tandem repeat (MIRU-VNTR) analyses. Their conclusions confirm the presence of the abovementioned strain types but clearly state that multiple genotyping techniques targeting different genetic markers are necessary to better discriminate against the homogeneous MAP population. This statement was further confirmed by Fritsch et al. (11), when reporting a directed epidemiological connection between wild red deer and farmed cattle. Only through a combination of two common SSR profiles, nine MIRU-VNTR patterns and nine IS900-RFLP patterns, it was possible to identify 17 different genotypes (11). Thus, taking into account that different genotypes can coexist within herds or in different species within the same habitat (11, 12), an additional epidemiological transmission study identified 15 MAP genotypes across multiple hosts by MIRU-VNTR analysis (13).

The first genome-wide single-nucleotide polymorphism (SNP) analysis of 141 global MAP isolates provided a greater resolution than the previous genotyping methods and confirmed a restricted genetic diversity with a low substitution rate (14, 15). This study, together with the dropping costs of the whole-genome sequence (WGS) analysis, paved the way for further MAP epidemiological studies (16–20).

A study carried out at the Stelvio National Park, a large protected area in the North of Italy, investigated the prevalence of the paratuberculosis in the red deer population and the genotype of the associated MAP (21). All isolates recovered from this latest study were type II and shared the same VNTR/SSR loci profile, suggesting a possible clonal infection.

In the present study, to further investigate the genetic diversity of this bacterium within the Stelvio National Park, we analysed the genomes of 15 MAP field isolates collected in a time range of 10 years using two different whole-genome sequence (WGS) methodology, a *de novo* assembly, and a reference-based approach. Furthermore, a comparative genomics analysis including MAP isolates from across the globe was carried out. Due to the low mutational rate (i.e., highly conserved genome) and in light of the disadvantages of the classical genotyping methodologies mentioned above, we believe that a combination of different next-generation sequencing (NGS) experimental approaches can benefit this field.

## 2 Materials and methods

### 2.1 Study area and sampling

The red deer study population analysed in this study inhabits the northwestern part of the Stelvio National Park, within the Province of Sondrio, central Italian Alps (46°28 0″N, 10°22′0″E). The population includes approximately 1,200 animals according to the annual counts, and their spatial distribution extends over 27,900 ha between 1,200 and 3,850 m a.s.l. (22). Year-around movements of individually marked deer and landscape features (ridges, valleys) support an absent or sporadic frequency of connection with other red deer populations (23). Sampling was carried out during the official culling plants within a temporal window of 10 years and was officially authorised by the “Istituto Superiore per la Protezione e la Ricerca Ambientale” (ISPRA) and the Italian Ministry of Environment (Prot. 48585/T-A25-Ispra).

*Mycobacterium avium* subsp. *paratuberculosis* field isolates herein analysed were recovered from faeces or intestines according to the procedure reported in the OIE Manual (24). Part of the isolates herein analysed have already been examined and resulted in all type II (type C) with the same VNTRs/SSRs profile (21), but in this case, we included all the isolates we were able to recover from our collection for a total of 15 isolates.

In addition to these field isolates, for phylogenetic and comparative genomics analysis, eight more MAP strains isolated from red deer worldwide were included in our study. According to their data availability, either raw reads (for the MAPMRI dataset) or complete genomes (for DT3 and K10 strains) were downloaded from the NCBI SRA database (Table 1).

**Table 1:** List of *Mycobacterium avium* field isolates used in this study.

Strain name	Isolation date	Host	Geographic origin	Accession number
M01	2016	*Cervus elaphus*	Italy	This study
M02	2009	*Cervus elaphus*	Italy	This study
M03	2016	*Cervus elaphus*	Italy	This study
M04	2013	*Cervus elaphus*	Italy	This study
M05	2020	*Cervus elaphus*	Italy	This study
M06	2020	*Cervus elaphus*	Italy	This study
M07	2020	*Cervus elaphus*	Italy	This study
M08	2020	*Cervus elaphus*	Italy	This study
M09	2020	*Cervus elaphus*	Italy	This study
M10	2019	*Cervus elaphus*	Italy	This study
M11	2019	*Cervus elaphus*	Italy	This study
M12	2013	*Cervus elaphus*	Italy	This study
M13	2013	*Cervus elaphus*	Italy	This study
M14	2012	*Cervus elaphus*	Italy	This study
M15	2012	*Cervus elaphus*	Italy	This study
MRI063	2005	*Cervus elaphus*	Germany	ERR037390
MRI064	2005	*Cervus elaphus*	Germany	ERR037391
MRI083	NA	*Cervus elaphus*	The Netherlands	ERR037959
MRI086	NA	*Cervus elaphus*	Czech Republic	ERR037962
MRI090	NA	*Cervus elaphus*	Argentina	ERR037966
MRI160	NA	*Cervus elaphus*	New Zealand	ERR248986
DT3	NA	*Cervus elaphus*	Great Britain	GCA_000240445.2
K10	NA	*Bos taurus*	United States	GCF_000007865.1

### 2.2 Sequencing, *de novo* assembly, and annotation

Total DNA was extracted from a pure culture of 15 *Mycobacterium avium* isolates, according to the procedure described in Bolzoni et al. (17). The DNA was then sequenced independently with an Illumina NextSeq platform, producing paired-end reads of 150 bp. Raw read quality was evaluated with FastQC (25), and the reads were filtered using Trimmomatic v0.39 (26) with the following parameters: CROP:140 HEADCROP:25 SLIDINGWINDOW:4:25 AVGQUAL:25 MINLEN:36.

In parallel, the raw reads of the ERR0 dataset downloaded from the NCBI SRA database were subjected to quality check and reads trimming as well. Due to the shorter read length of this MAPMRI dataset (76 bp average), the trimming parameters were modified as follows: CROP:65 SLIDINGWINDOW:4:20 AVGQUAL:25 MINLEN:36.

The trimmed reads were then *de novo* assembled using SPAdes v3.15.4 (27) with default parameters and k-mers set to 21, 33, 55, and 77, with the exception of the MAPMRI dataset, where a k-mer length of 77 was not used. The assembly statistics were evaluated using QUAST 5.0 (28). Structural and functional annotations were performed with Prokka v1.14.5 (29), using the pre-built Prokka database, further supported by the MAP K10 annotation as the Reference genome. For both DT3 and K10 strains, already assembled, all of the above steps were skipped.

### 2.3 Pangenome and comparative analysis

For whole-genome comparison, we adjusted the pipeline used in Turco et al. (30). Briefly, all the genomes listed in Table 1 were reordered with MAUVE (31) towards K10 set as the reference genome and then aligned using the *progressiveMAUVE* algorithm with default parameters (31). The average nucleotide identity (ANI) was calculated on the entire genomes with MUMMER v3.1 within the pyANI script (32). Prokka results in a Gff3 format were given as input to Roary v1.7.7 (33) in order to get a core-genome alignment used to construct a maximum 133 likelihood (ML) phylogenetic tree with RAxML-HPC, setting the GTRCATI algorithm as the substitution model and 1,000 bootstraps (34).

The tree was visualised with FigTree v.1.4.4^1^ and edited with Inkscape v0.92.^2^

A pangenome analysis was performed with Anvi’o v.7 (35), providing Prokka results in a GenBank format as input files, using NCBI BLASTP for amino acid sequence similarity search, the default minbit heuristic set to 0.5, and the MCL inflation parameter set to 10. Besides Prokka, all the genomes were further annotated using NCBI’s Clusters of Orthologous Groups (COG) database (36) and Pfam (37). Bins for single-copy core gene (SCG), accessory, and singletons were retrieved and further analysed.

### 2.4 Identification of effector genes

*Mycobacterium avium* subsp. *paratuberculosis* effector genes were downloaded from the virulence factors of pathogenic bacteria (VFDB, http://www.mgc.ac.cn/VFs/main.htm, Accessed on March 1, 2023) and blasted on the MAP assemblies under comparison using NCBI BLASTn. Using an *in-house* python script, each effector was considered present if it showed a minimum of 70% of identity and at least 70% of query coverage. The results were then used to create a presence/absence binary matrix that was plotted as a heatmap of the presence/absence using the pheatmap package v1.0.12 (38) within the R environment v3.4.4.

### 2.5 Reference-based assembly

A reference-based assembly was also carried out to better evaluate the genetic diversity among the MAP isolates under comparison and, thus, to identify the possible genomic regions that did not map on the reference genome and that are characteristic of these MAP isolates. For this purpose, the trimmed raw reads were aligned towards the K10 genome using BWA aligner v0.7.12 (39) with default parameters. Once obtained the alignment BAM files, the mapped and unmapped reads were retrieved with SAMtools v1.13 (40) and the SNP variants called with SAMtools and BCFtools (40), setting a minimum base quality of 50 and a minimum mapping quality of 30, as suggested by Bryant et al. (14). These SNPs variants were used to build an ML phylogenetic tree using RAxML-HPC, with GTRCATI algorithm as the substitution model and 1,000 bootstraps (34) and to extract the final consensus sequence from each isolate. The ANI of these consensus sequences was calculated with MUMMER v3.1 within the pyANI script (32). Moreover, as mentioned above, the unmapped reads were further *de novo* assembled with SPAdes v3.15.4 (27) and annotated with Prokka v1.14.5 (29). CDS were further inspected to identify possible deer-specific features and other bacteria origins, mapping them against the online NCBI database. All the calculations have been carried out at Cineca in the framework of the ELIXIR-IT HPC@CINECA program (41) and on the Tuscia-DIBAF HPC center.

## 3 Results

### 3.1 DNA sequencing and reads trimming

For the *in-house* sequenced samples, total DNA was extracted from each sample, as already reported (17) in the protocol and sequenced with an Illumina NextSeq platform using a paired-end sequencing method, while the MAPMRI dataset was downloaded from the SRA NCBI database (Table 1). For both datasets, the raw reads number, before and after trimming, together with their length, are shown in Supplementary Table S1. The stringency of the trimming parameters was chosen according to the FastQC results, and the quality of reads was re-checked after trimming. The trimmed raw reads were used for two distinct pipelines: a *de novo* assembly-based and a reference-based assembly method.

### 3.2 *De novo* assembly and features annotation

Trimmed reads assembled with SPAdes v3.15.4 (27) yielded a total number of contigs ranging from 461 (M07) to 2,374 (M15), with a total genome length spanning from 4,601,483 (M05) to 5,031,920 (M01) (Supplementary Table S2). The assembly QUAST statistics, shown in Supplementary Table S3, indicate an average GC content above 69% and the presence of several undetermined bases. Prokka annotation identified 4,734 CDS on average, with three rRNA, one tmRNA in all the samples, and ranging from 55 to 58 tRNAs (Supplementary Table S2). Furthermore, among the identified CDS, an average of 1,237 were shown to have hypothetical functions and 3,428 were instead codified for a feature related to K10 annotation.

### 3.3 Genome alignment and average nucleotide identity

For a whole-genome comparison to identify possible rearrangement and insertions/deletions (Indels) events, all the contigs were reordered towards the K10 genome and further aligned as shown in Supplementary Figure S1. The coloured blocks represent conserved genomic regions aligned to K10, either in forward (above the centre line) or reverse (below the centre line) orientation. Despite the overall generic conservation, there are blocks with a lower similarity profile that are coloured in white within the blocks. Completely missing coloured blocks indicate a deletion in that region, while regions outside the blocks may represent sequence elements specific to a particular genome, like in the cases of M01 and M04 (Supplementary Figure S1).

**Figure 1:**
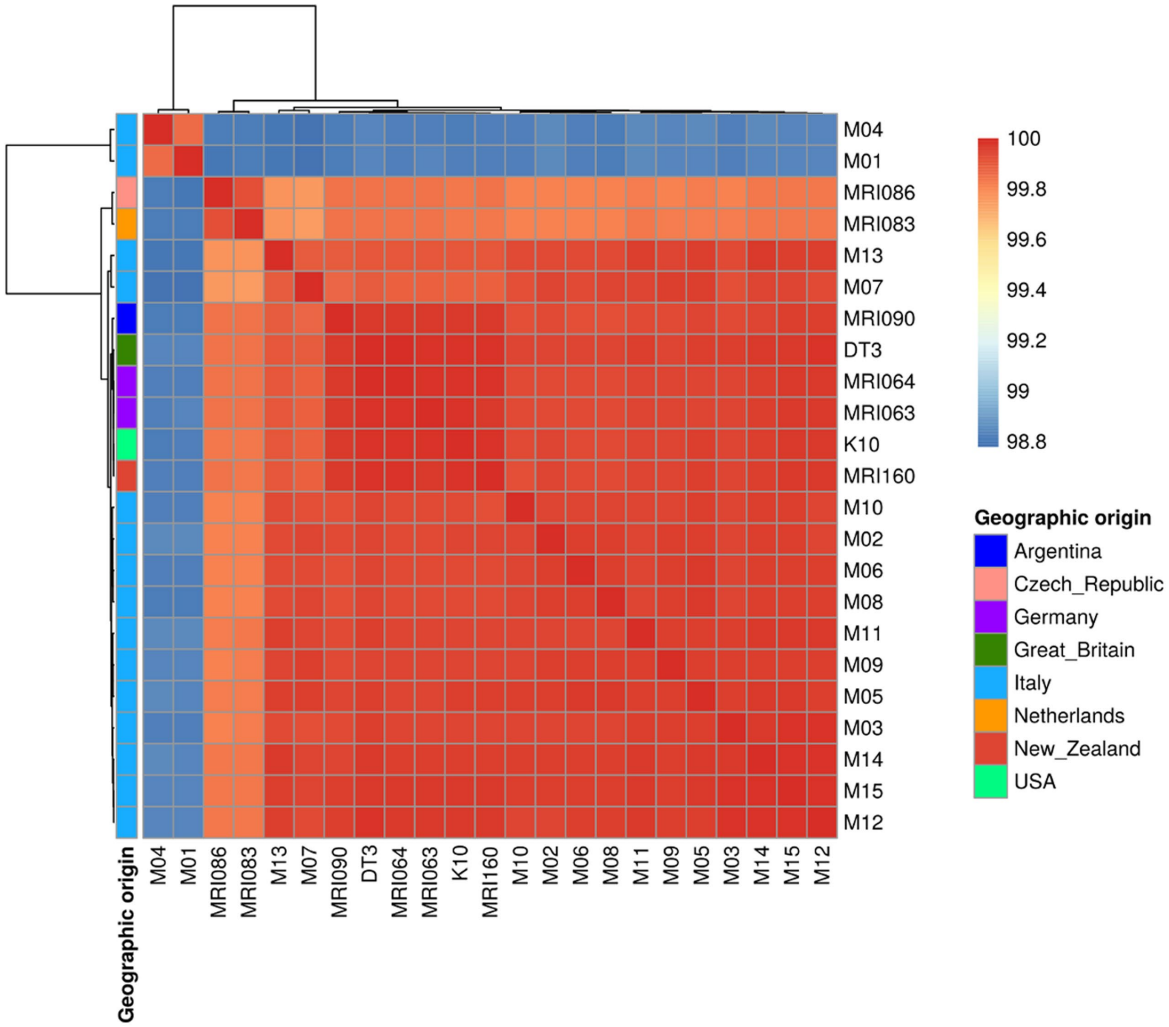
A Heatmap showing the Average Nucleotide Identity (ANI) among the MAP strains under comparison indicated as a percentage of identity ranging from 98.8 to 100%. The geographic origin of each sample is shown as well.

The colour gradient of the ANI heatmap indicates an ANI spanning from 98.8 up to 100% (Figure 1). The majority of the Italian field isolates are quite similar to each other, clustering together with the rest of the foreigner isolates and reference strains K10 and DT3 (Figure 1). However, two field isolates (M01 and M04) were clustered on their own with the lowest percentage of 98.8 with the reference K10 genome (see below). All the other isolates are grouped together, except for MRI086 from the Czech Republic and MRI083 from the Netherlands clustered apart, with an average of identity of 99.7%.

### 3.4 Pangenome analysis

**Figure 2:**
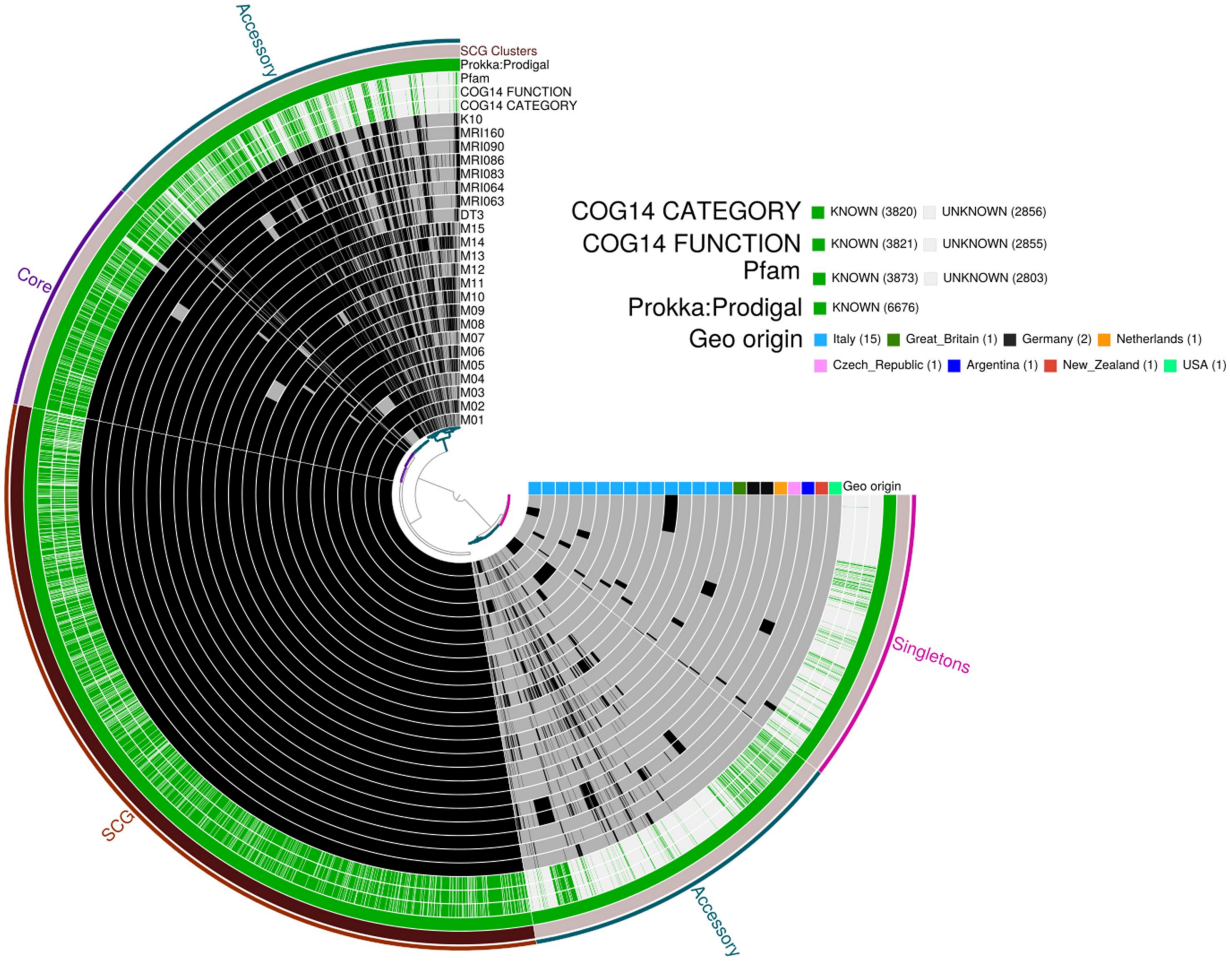
Pangenome analysis performed with Anvi’o. From the outermost to the innermost: (*i*) coloured bins indicating the accessory genes, the core genes, Single-Copy core Genes (SCG), and singletons; (*ii*) Single-Copy Gene cluster; (*iii*) Prokka: Prodigal, Pfam, COG functional annotation, and COG category annotation; and (*iv*) individual genomes organised regarding their phylogenetic relationships, with the dark colour indicating the presence of the gene cluster and the light colour its absence. Geographic origin and biovar affiliation are indicated as well.

**Figure 3:**
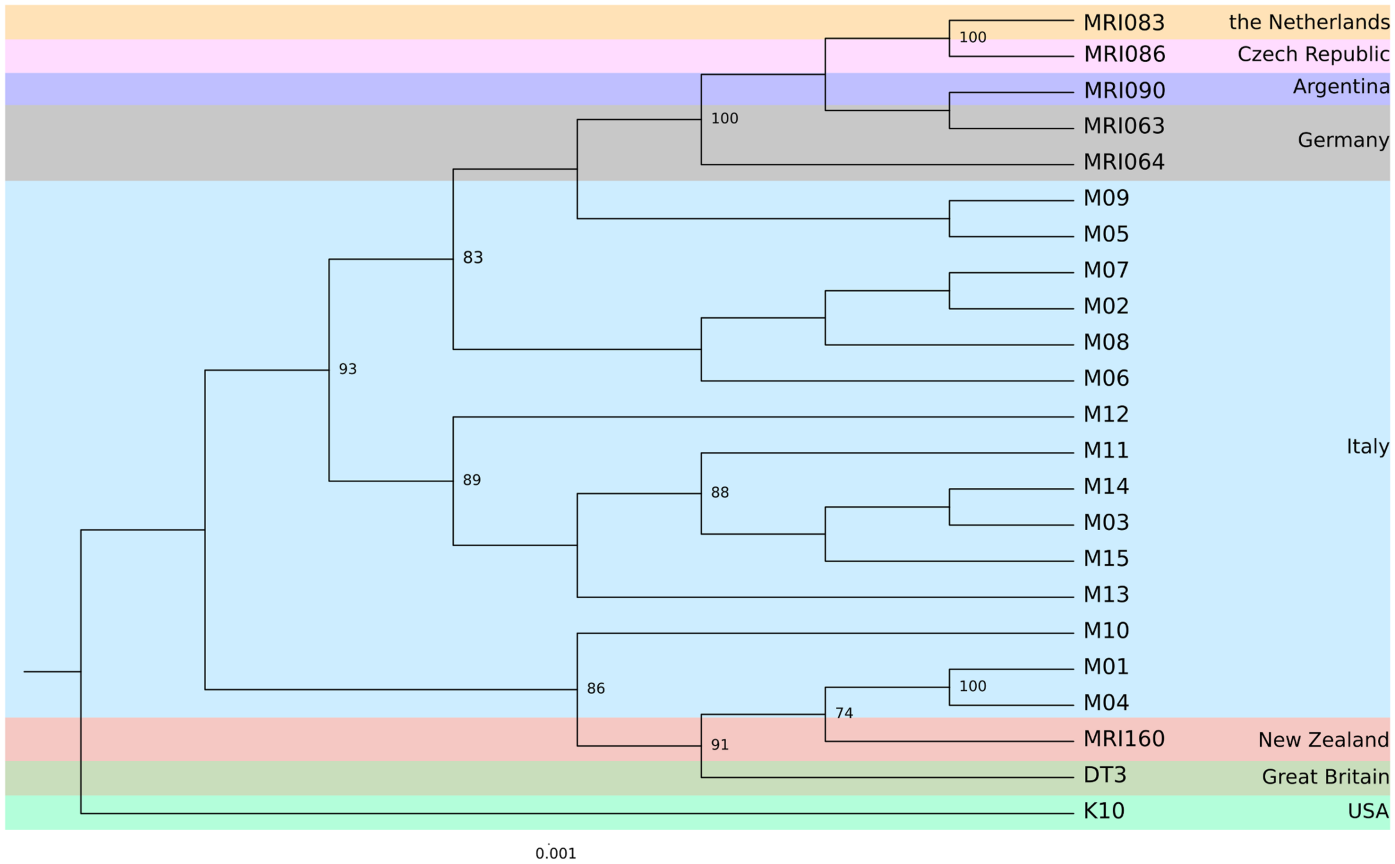
Maximum Likelihood (ML) phylogenetic tree based on the core genome alignment identified by Roary among the MAP strains from eight different geographic areas. The number of bootstraps above 70 is indicated as node labels.

The pangenome analysis identified a total of 107,458 genes arranged in 6,964 gene clusters (Figure 2). Among those, 870 gene clusters are defined as “core genome,” shared between all the MAP isolates, and represent 37% of the total genome. The core genome was then used to build the phylogenetic tree shown in Figure 3. In line with the results shown in Figure 1 for the whole-genome comparison, the core genome confirms the different genomic characteristics of the isolates M01 and M04, which cluster on a separate branch. These two isolates show decreasing similarity to MRI160 from New Zealand, DT3 from Great Britain, and M10 from Italy. All of the remaining isolates belong to a single branch that includes the samples from Germany, Argentina, Czech Republic, and the Netherlands, which are different only from K10 (Figure 3).

**Figure 4:**
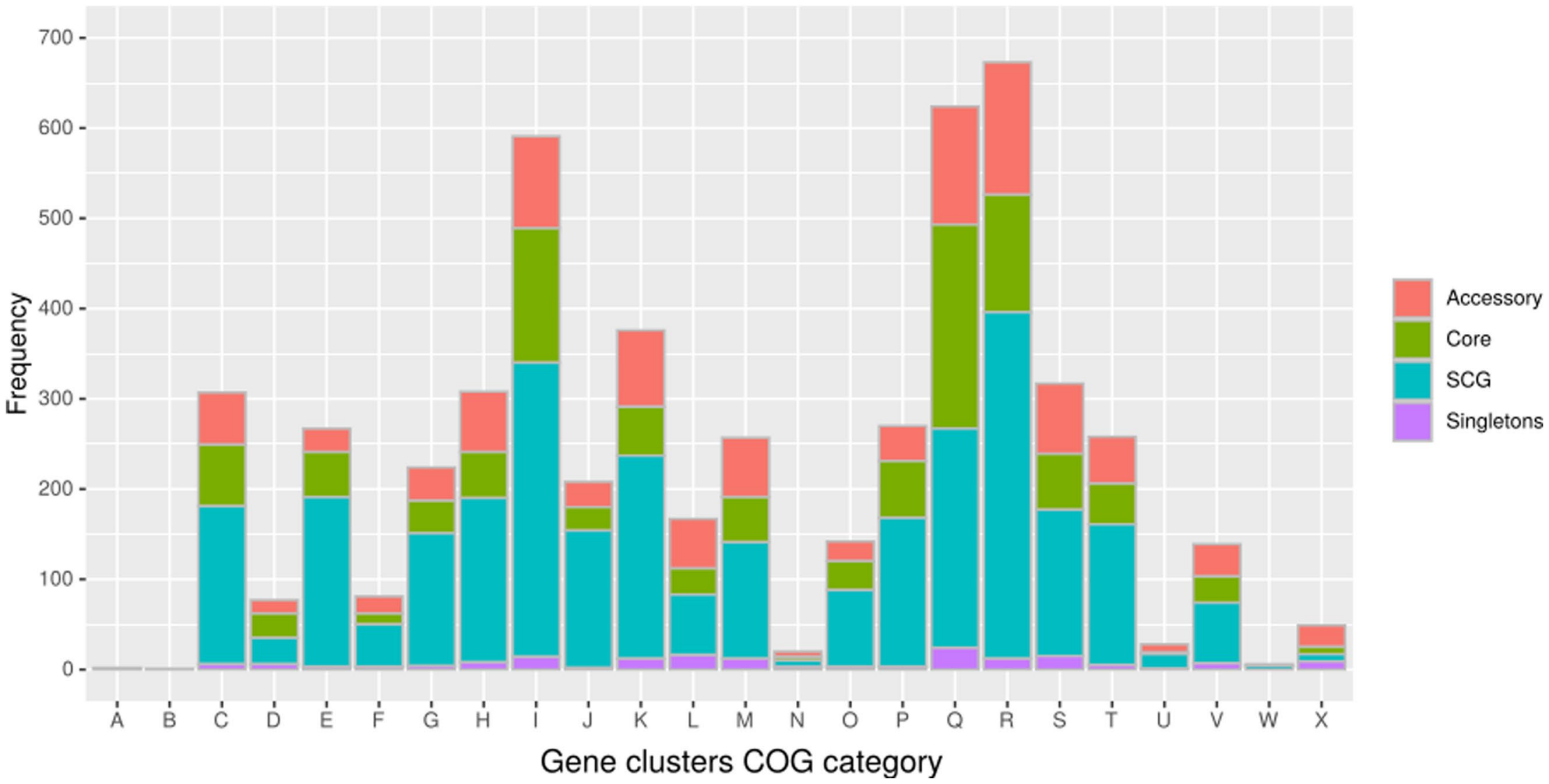
Distribution of the gene clusters identified by Anvi’o among the different Cluster of Orthologous groups (COG). **(A)** RNA processing and modification (not used for prokaryotic COGs); **(B)** chromatin structure and dynamics; **(C)** energy production and conversion; **(D)** cell cycle control and mitosis; **(E)** amino acid metabolism and transport: **(F)** nucleotide metabolism and transport; **(G)** carbohydrate metabolism and transport; **(H)** coenzyme metabolism; **(I)** lipid metabolism; **(J)** translation; **(K)** transcription; **(L)** replication and repair; **(M)** cell wall/membrane/envelope biogenesis; **(N)** cell motility; **(O)** post-translational modification, protein turnover, and chaperone functions; **(P)** inorganic ion transport and metabolism; **(Q)** secondary metabolites biosynthesis, transport, and catabolism; **(R)** general functional prediction only (typically, prediction of biochemical activity); **(S)** function unknown; **(T)** signal transduction; **(U)** intracellular trafficking and secretion; **(Y)** nuclear structure (not applicable to prokaryotic COGs); and **(Z)** cytoskeleton (not applicable to prokaryotic COGs).

In the pangenome analysis (Figure 2), 2,827 core gene clusters were present in one copy per genome (SCG, Single Core Genome). These elevated numbers indicate high genomic similarity with a low evolutionary rate. Besides the 2,336 accessory gene clusters, 931 gene clusters were defined as singletons, peculiar for one single isolate (Supplementary Figure S2A). A COG functional category could not be assigned to 2,995 gene clusters, 161 from the core genome, 515 SCG, 808 singletons, and 1,511 accessory gene clusters (Supplementary Figure S2B). On the contrary, the majority of the gene clusters belonged to COG category “R” of general functional prediction only (typically, prediction of biochemical activity), category “Q” of secondary metabolites biosynthesis and category “I” of lipid metabolism, with the rest of the singletons spread among the different categories (Figure 4). Interestingly, the isolate with the highest number of singletons was M11, with 250 singletons, followed by M01, M05, and M15 with 157, 74, and 72 singletons, respectively (Supplementary Table S4). Despite most of the singletons being annotated as hypothetical proteins without COG category assignment, other singletons belonged to COG “I,” “Q,” “R,” and “S” categories (Supplementary Figure S3). No COGs have been observed for category “W” (extracellular structures) and very few COGs for category “N” (cell motility). Among these, of particular relevance are the singletons annotated as K10 MCE family protein (COG category “M”) and found in M01, M09, and M11, one singleton belonging to the K10 PPE family protein (COG category “S”), and both families associated with virulence, as well as transposase, DNA invertase that could contribute to this genome plasticity (Supplementary Table S4). Other singletons were annotated as TetR/AcrR transcriptional regulator family, besides several oxidoreductase, hydrolase, and recombinase. Overall, the Pfam annotations were comparable to those from COG and Prokka.

A relatively small number of genes (40) was present only in M01 and M04 samples, whose most frequent COG categories are “Energy production and conversion” and “Defence mechanisms” with eight and seven genes, respectively.

### 3.5 Virulence-related features

**Figure 5:**
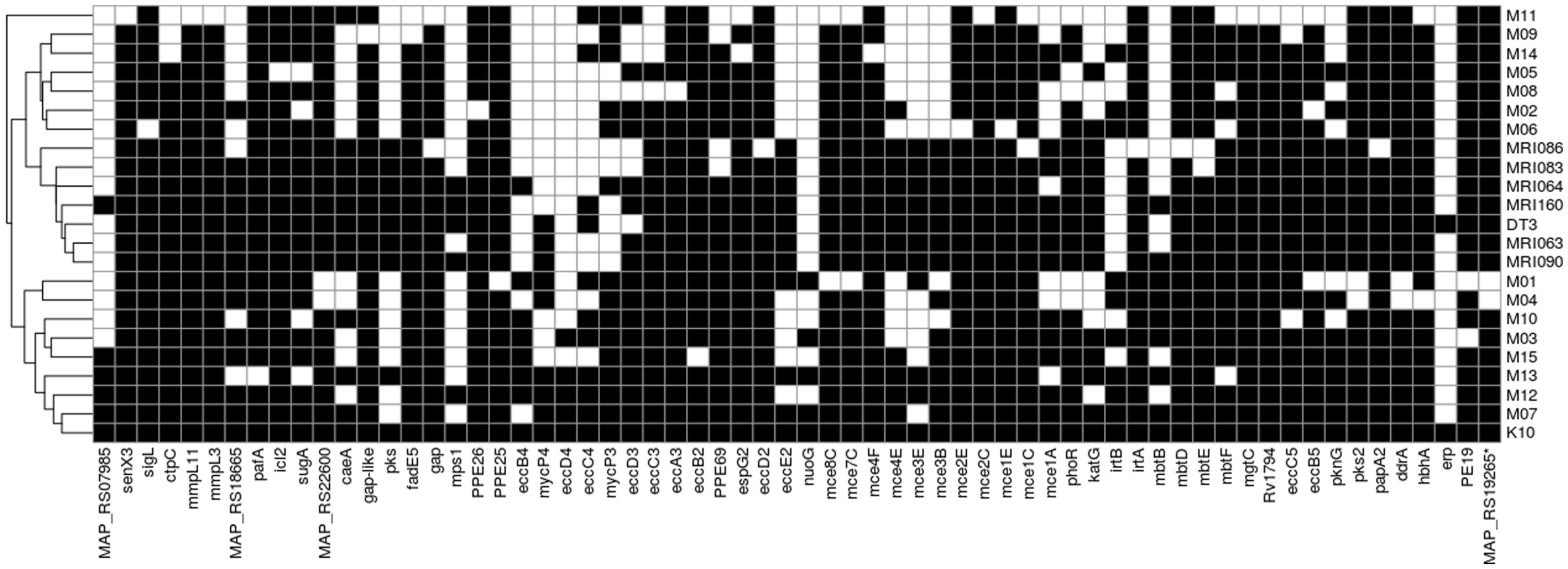
Distribution of K10 virulence-related features among the MAP strains. The black block indicates the presence of the feature retrieved by BLAST alignment, while the white block indicates its absence.

A total of 199 MAP virulence-related features (i.e., effector genes) from K10 were downloaded from the VFDB database and blasted on the MAP isolates under comparison. A total of 131 features were found in all the isolates, mostly related to immune modulation, nutritional or metabolic factors, effector delivery system, adherence, regulation, ABC transporters, and the MCE family proteins (Supplementary Table S5). Despite four effectors that were found only in K10 (effector delivery system RS21760, mycP2, mycP5, and PPE4), there is no particular virulence factor related only to the MAPs isolated from red deer (Figure 5). Interestingly, M01 and M04 are the only two isolates where psk2, ddrA, MAP_RS22660, and MAP_RS1926 were not present, confirming the peculiarity of these two isolates with respect to MAPs herein isolated.

### 3.6 Reference-based assembly

Independent to the *de novo* assembly pipeline described above, the trimmed reads were aligned to the reference K10 genome for a reference-based assembly using BWA with default parameters. After variant calling and SNP filtering according to base quality, mapping quality, and read support, a ML phylogenetic tree was built on the concatenated SNPs (Supplementary Figure S4). The high clonal level of the MAP isolates reconstructed with this method appears immediately clear, with all the samples clustering together, except for the M01 and M04 that in the *de novo* assembly already appeared different. To further verify this high genetic similarity, the consensus sequence was extracted from the aligned BAM file using SAMtools and BCFtools. The sequences were then aligned to each other and to the assembled K10 and DT3 to perform an ANI analysis, as shown in Supplementary Figure S5. Overall, the percentage of identity was higher than 99%, with the lowest similarity with M01 and M04, in line with the *de novo* assembly results previously presented. The F57 sequence was not found in these two last isolates.

Furthermore, the K10 unmapped reads from each sample were *de novo* assembled and annotated with Prokka database (Supplementary Table S6). Only two or three CDS (annotated mostly as hypothetical proteins) were identified, starting from less than 10 contigs for almost all the MAP isolates, while M01, M04, and M15 showed 580, 608, and 307 CDS, respectively. In line with previous results that showed the similarity between M01 and M04, 569 out of 580 CDS from M01 resulted to be 100% identical to the ones from M04. When blasted on the NCBI database, the major part of the common CDS (almost 60%) resulted similar to *M. avium* subsp. *hominissuis*, 28% to *M. avium* and only 0.44% to *M. avium* subsp. *paratuberculosis* (Supplementary Table S7). To confirm whether the two isolates belong to *M. avium* subsp. *hominissuis*, all the isolates were mapped against the *M. avium* subsp. *hominissuis* reference sequence (MA104—https://www.ncbi.nlm.nih.gov/datasets/taxonomy/243243/), following the same protocol used for K10 reference-based approach. The percentage of unmapped reads was 5.1 and 2% for M01 and M04, respectively; for the other isolates, the minimum was 7% for M06 and the maximum was 10.7% for M15 (Supplementary Table S6). Two of the remaining 11 CDS were related to K10 TauD/TfdA dioxygenase and HNH endonuclease (and indeed present as well in K10) and nine hypothetical proteins (SM1). When using BLAST from the NCBI database, the nucleotide sequence of these 11 CDS resulted more similar to *M. avium* subsp. *hominissuis*, besides few CDS related also to *M. avium* subsp. *paratuberculosis* and still with only hypothetical annotation. A total of 33 CDS from M04 were not present in both M01 and K10, even if 11 of them were annotated as spirocyclase, MMPL family transporter (considered a virulence factor in MAP) [Vilijoen et al., 2017 (42)], cytochrome P450, SDR family NAD (P)-dependent oxidoreductase, aldehyde dehydrogenase, twice TetR/AcrR, CbbQ/NirQ family protein, TNT antitoxin, and alpha/beta hydrolase related to K10 (SM2). When blasted, the nucleotide sequences of these 33 CDS appeared to be related to *M. conspicuum*, *M. intracellulare*, and *M. avium* subsp. *hominissuis*, confirming the peculiarity of sample M04. Plus, the M04 singletons identified by Anvi’o were also found among the unmapped CDS. None of the above CDS coming from both M01 and M04 were in common with M15. M15 singletons from Anvi’o analysis, instead, were found in the unmapped CDS as well, mostly related to transposase, oxidoreductase, heavy metal translocase, and teichoic acids export ATP-binding protein (Supplementary Table S4). Among the other dataset, MRI083 and MRI086 were the two with the highest number of unmapped contigs, codifying for 107 and 108 CDS, respectively (Supplementary Table S6).

## 4 Discussion

*Mycobacterium avium* subsp. *paratuberculosis* (MAP) is a microorganism characterised by an extremely slow growth rate both in a controlled environment (*in vitro*) and within living organisms (*in vivo*). One of the consequences of this slow pace is a low mutation rate and the extremely well-conserved and closed core genome (14, 15, 17, 20). In the field of MAP epidemiology, traditional population genetic and diversity analysis methods such as VNTR or SSR analysis have currently been largely supplanted by WGS approaches. These new methods offer significantly higher discriminatory power, even in populations characterised by extremely low diversity, such as *Mycobacterium bovis* (43) and MAP (17, 44).

In this study, we have investigated the genomic structure of *Mycobacterium avium* isolated from red deer inhabiting the Stelvio National Park using WGS. Notably, a prior investigation focussing on a subset of the analysed field isolates had assessed the MAP population structure using minisatellite and microsatellite loci revealing only one distant profile (21), suggesting a clonal origin of infection.

Our WGS analysis, employing two different approaches—a *de novo* assembly and a reference-based reads alignment on MAP K10 isolate, largely confirms this initial observation, highlighting the presence of a single major clade. Moreover, our results unveiled the existence of other field isolates (M01 and M04) belonging to a different subspecies within the *Mycobacterium avium* species, the *Mycobacterium avium* subsp. *hominissuis*. Further analyses demonstrated a strong similarity between these two isolates markedly different in genome content from the other MAPs. Despite a substantial time gap between the isolation of these two field isolates, we hypothesise that there was no epidemiological link between the two infections. To reinforce this classification, both isolates lacked the F57 sequence, one of the most specific MAP markers (2). The presence of *Mycobacterium avium* subsp. *hominissuis* has previously been documented in red deer in Austria and Hungary (45, 46), Specifically, in Austria, instances were recorded during 2001–2002, while Rónai et al. (46) reported isolates collected between 2006 and 2015. This particular subspecies of *Mycobacterium avium* is considered an environmental bacterium, often found in water, soil, dust, or straw, and its primary hosts include pigs and humans (47).

The successful utilisation of both *de novo* assembly and reference genome alignment for exploring diversity within the MAP population has already been documented (18, 48). Notably, we believe that both approaches should be used to investigate the MAP population structure of field isolates apparently coming from the same outbreak or very close ones. While the *de novo* method circumvents biases related to indels or genetic translocations, the less computationally intensive reference genome alignment is appropriated for reconstructing and comparing core genomes. Consequently, building epidemiological trees based on SNP analyses of the core genome remains the predominant approach in evaluating potential epidemiological links within the MAP field, due to its notably low mutation rate (14, 17, 19, 20).

The analyses of the singletons of our red deer isolates revealed that the majority of COGs belong to “S” category (proteins with unknown function), followed by “I” category (lipid transport and metabolism), “Q” category (secondary metabolites biosynthesis, transport, and catabolism), “R” category (general function prediction only), and “K” category (transcription). In a recent study, Lim et al. (18), analysing different types of MAP (type I, type II, and type III), observed that the main part of COGs fell into the categories S (function only) and R (general function prediction only), and among the well-characterised genes, the major part mapped into categories was related to metabolism, in particular “Q,” “I,” and “K,” eventually mirroring what we have observed in the present study for MAP type II. This observation suggests a possible common trend for all MAP isolates independent of the type analysed.

Focussing on the differences between the red deer isolates herein analysed and the K10 genome, it is worth noting that the absence in all our samples of RS21760, mycP2, mycP5, and PPE4. Myc P2 and P5 genes codify for two predicted serine proteases with unknown function (49), and the role of effector delivery system RS21760 is still unknown. The PPE proteins have conserved proline (P) and glutamic acid (E) residues in their N-terminal sequences and are involved in outer membrane nutrient transport and host–pathogen interaction or immune evasion (50). In more detail, PPE4 is important for mycobactin-mediated iron acquisition (50), and since it is absent in all isolates herein presented but K10, we could hypothesise that its presence in K10 is due to the adaptive stress for *in vitro* multiple passages.

We also observed differences in the two M01 and M04 isolates, which clustered differently with respect to the others. All the red deer samples, including K10, have in their genome the virulence-related features psk2, ddrA, MAP_RS22660, and MAP_RS1926 absent in M01 and M04. Moreover, the two field isolates M01 and M04 did not show in their genomes pks2, MAPRS 22660, MAPRS 19265, and ddrA. In more detail, ddrA gene codifies for a protein which is part of the ABC transporter complex DrrABC involved in doxorubicin resistance (51) and of the transport and synthesis of phthiocerol dimycocerosate (PDIM) (52), a virulence factor of *Mycobacterium tuberculosis* (53); however, the pks2 gene codifies for a protein involved in sulpholipid biosynthesis (54). No information is currently available for the products of genes MAPRS 22660 and MAPRS 19265.

More importantly, in M01 and M04, the F57 sequence, one of the most specific MAP markers (2), is absent. This evidence is in line with the alignment results that assign M01 and M04 to *M. avium* susbsp. *hominissuis.*

## 5 Conclusion

The use of WGS in the field of MAP epidemiology confirms the clonal nature of the paratuberculosis outbreak present in the red deer inhabiting the Stelvio National Park, demonstrating the presence of a single major clade in a time range of a decade. We also identified the presence of two isolates belonging to *M. avium* subsp. *hominissuis* in the red deer population. Even if the reduced number of samples did not permit us to make any hypotheses about the origin of the infection and the evolution within the red deer population inhabiting the Stelvio National Park, it is worth noting these two isolates being isolated in two different locations of the Stelvio National Park and in different years.

In future investigations, it will be pivotal to acquire additional metadata for red deer at the individual and population levels and on MAP isolates from livestock sharing summer pasture to make hypotheses about the origin and the evolution of the multi-host infection. A multidisciplinary approach incorporating molecular epidemiology and ecology into traditional infectious disease knowledge will improve the prevention measures in a semi-extensive production system and support decision-making on a poorly addressed topic.

## Data availability statement

The datasets presented in this study can be found in online repositories. The names of the repository/repositories and accession number(s) are: https://www.ncbi.nlm.nih.gov/, PRJNA986832. The python script used for the analysis of the effector genes is available on request to daniele.pietrucci@unitus.it.

## Ethics statement

This study uses strains obtained at the Istituto Zooprofilattico Sperimentale della Lombardia e dell’Emilia Romagna. The Istituto Zooprofilattico Sperimentale della Lombardia e dell’Emilia Romagna did not require the study to be reviewed or approved by an ethics committee because some field isolates have been obtained in the frame of Routine diagnostic activity at Istituto Zooprofilattico Sperimentale della Lombardia e dell’Emilia Romagna (faecal specimens). The others have been recovered from animals that have been culled for management purposes according to the official culling plan to reduce red deer density that has been authorised by Istituto Superiore per la Protezione e la Ricerca Ambientale (ISPRA), the Italian Ministry of Environment (Prot. 48585/T-A25-Ispra), in the Lombardy sector of the Park. Therefore, animals were not sacrificed for research purposes specific to this study.

## Author contributions

ST: Investigation, Writing – original draft, Writing – review & editing. SR: Methodology, Writing – review & editing. DP: Methodology, Software, Writing – review & editing AF: Methodology, Writing – review & editing. MM: Writing – review & editing, Conceptualization, Investigation, Writing – original draft. CL: Conceptualization, Writing – review & editing. CG: Writing – review & editing, Funding acquisition, Resources. GP: Writing – review & editing, Methodology. GC: Conceptualization, Data curation, Investigation, Software, Writing – original draft, Writing – review & editing. MR: Conceptualization, Data curation, Funding acquisition, Investigation, Project administration, Writing – original draft, Writing – review & editing.

## Footnotes

^1^ http://tree.bio.ed.ac.uk/software/figtree

^2^ www.inkscape.org
