# Supplementary material for: High clonality of Mycobacterium avium subsp. paratuberculosis field isolates from red deer revealed by two different methodological approaches of comparative genomic analysis
Source: Front Vet Sci. 2024 Feb 6;11:1301667. doi: 10.3389/fvets.2024.1301667 (PMC10876796; doi:10.3389/fvets.2024.1301667)
Supplement: Supplementary file 8 [file Data_Sheet_1.PDF]

Supplementary Figures

Article Title

High clonality of *Mycobacterium avium* subsp. *paratuberculosis* field isolates from red deer revealed by two different methodological approaches of comparative genomic analysis

Silvia Turco1, Simone Russo2, Daniele Pietrucci3, Anita Filippi2, Marco Milanesi3, Camilla Luzzago4, Chiara Garbarino2, Giorgia Palladini2, Giovanni Chillemi3,5*, Matteo Ricchi2*.

*Corresponding authors: gchillemi@unitus.it, matteo.ricchi@izsler.it

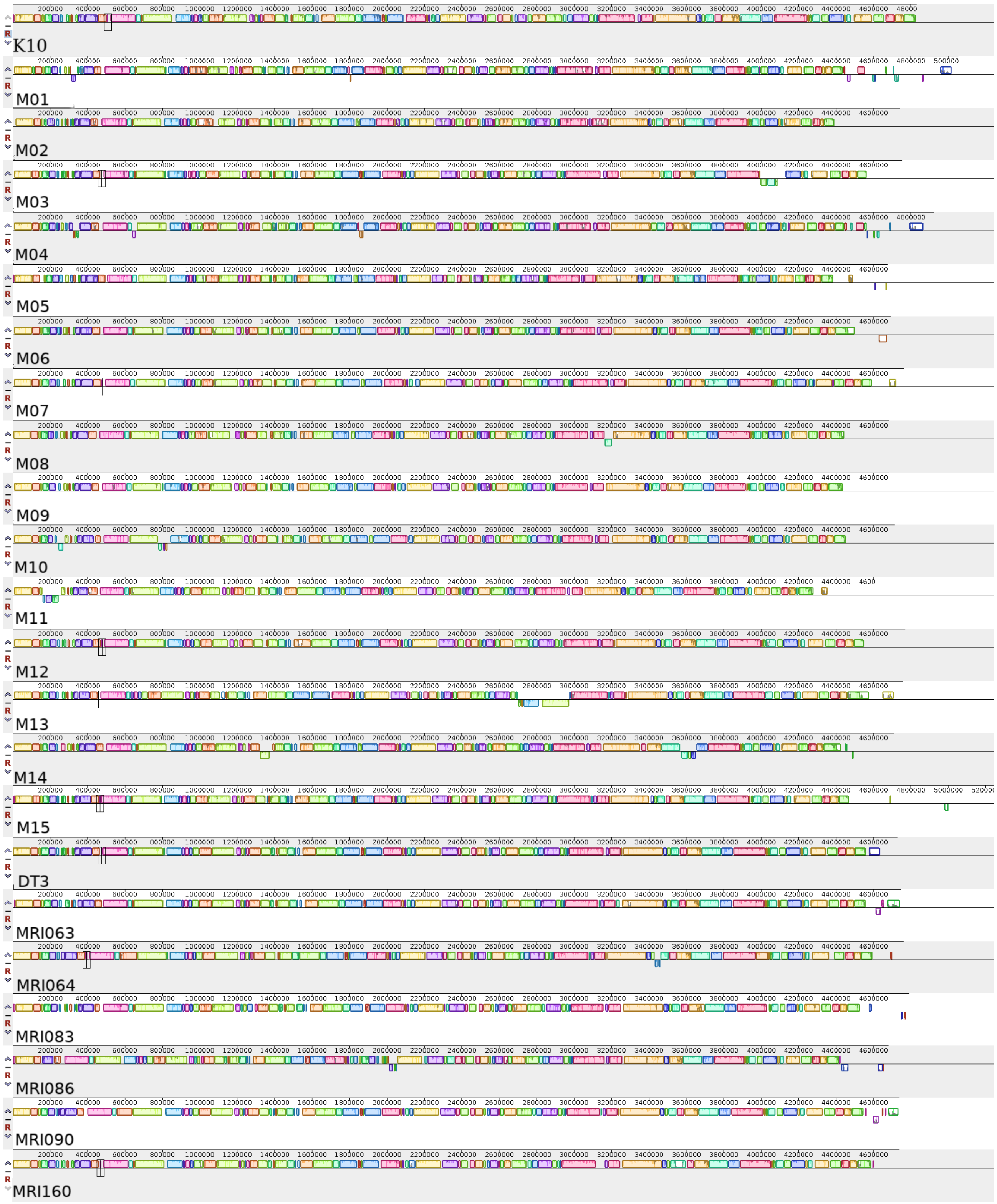

Figure S1: Mauve alignment of the *Mycobacterium avium* genomes, using K10 as Reference genome. The coloured blocks indicate synteny region, while the blocks with white indicate low similarity region.

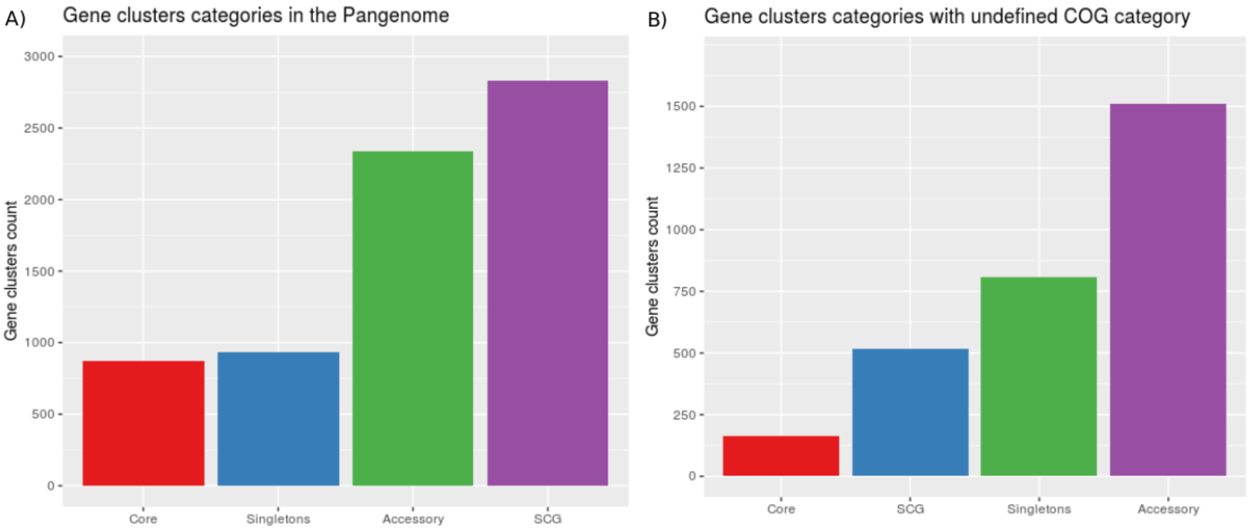

Figure S2: A) Counts of gene clusters among the different pangenome categories as Accessory, Core, SCG and singletons. B) Counts of gene clusters among the different pangenome categories as Accessory, Core, SCG and singletons with undefined COG category.

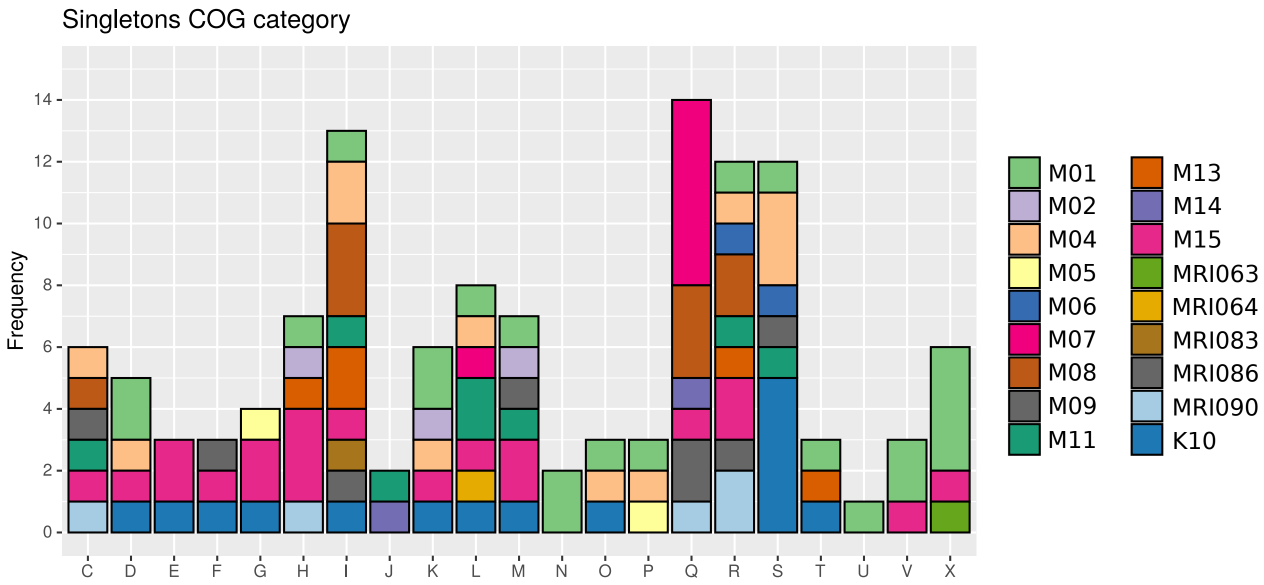

Figure S3: Distribution of the singletons gene clusters identified by Anvi'o among the different Cluster of Orthologous groups (COG). A: RNA processing and modification (not used for prokaryotic COGs); B: chromatin structure and dynamics; C: energy production and conversion; D: cell cycle control and mitosis; E: amino acid metabolism and transport: F: nucleotide metabolism and transport; G: carbohydrate metabolism and transport; H: coenzyme metabolism; I: lipid metabolism; J: translation; K: transcription; L: replication and repair; M: cell wall/membrane/envelope biogenesis; N: cell motility; O: post-translational modification, protein turnover, chaperone functions; P: inorganic ion transport and metabolism; Q: secondary metabolites biosynthesis, transport and catabolism; R: general functional prediction only (typically, prediction of biochemical activity); S: function unknown; T: signal transduction; U: intracellular trafficking and secretion;

Y: nuclear structure (not applicable to prokaryotic COGs); Z: cytoskeleton (not applicable to prokaryotic COGs).

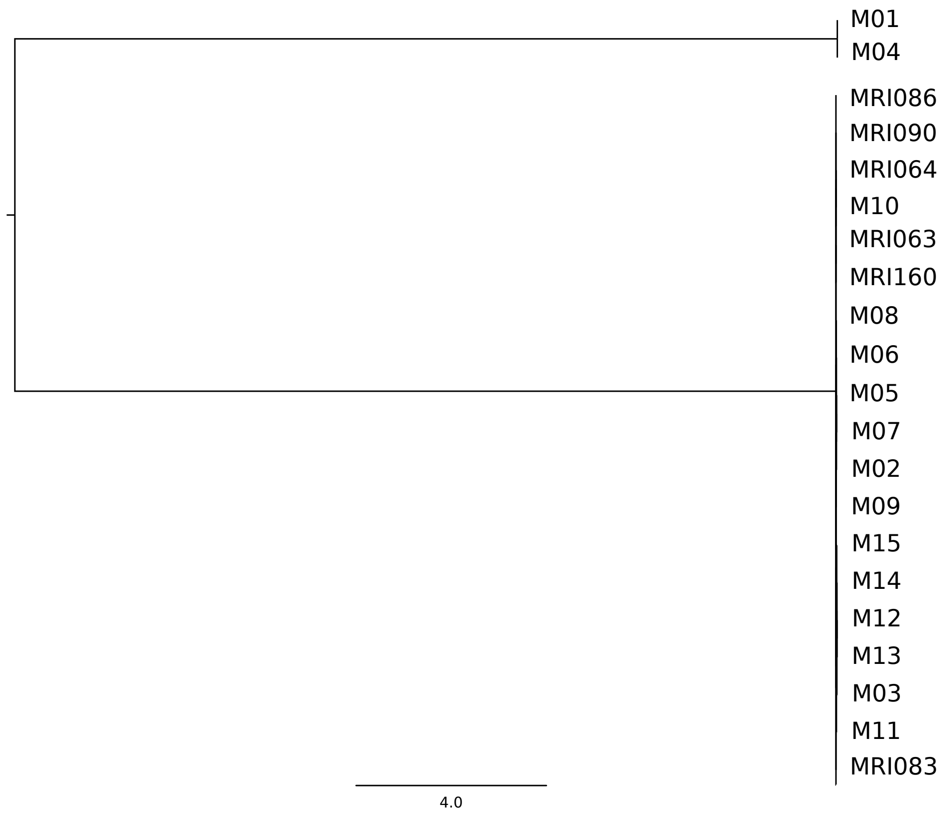

Figure S4: ML phylogenetic tree based on the core genome alignment of the MAP consensus sequences retrieved from the Reference-based assembly.

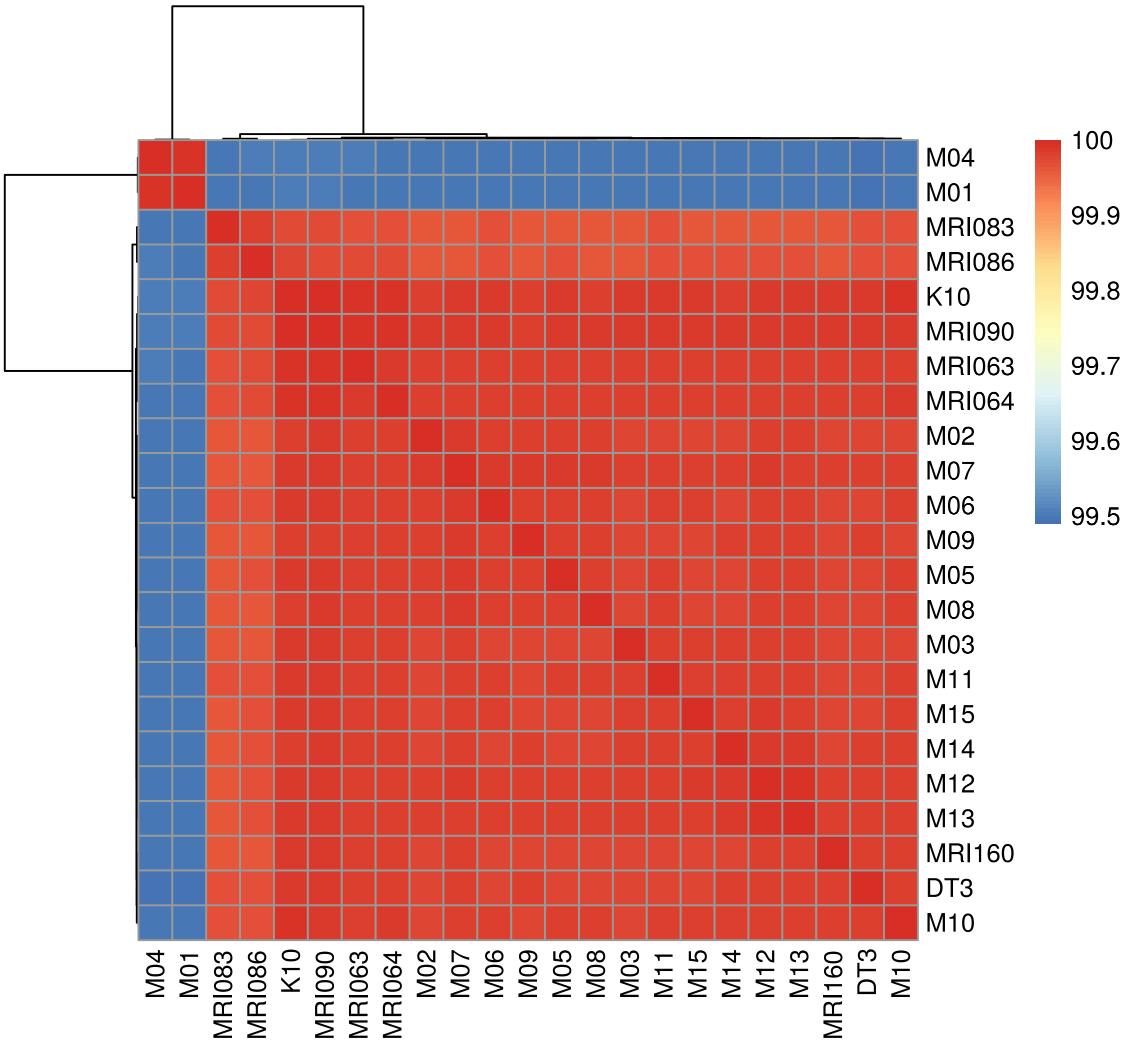

Figure S5: Heatmap showing the Average Nucleotide Identity (ANI) among the *Mycobacterium avium* genomes obtained through Reference-based assembly. The percentage of identity ranges from 98.8 to 100%. The geographic origin of each sample is shown as well.
