# Supplementary material for: High clonality of Mycobacterium avium subsp. paratuberculosis field isolates from red deer revealed by two different methodological approaches of comparative genomic analysis
Source: Front Vet Sci. 2024 Feb 6;11:1301667. doi: 10.3389/fvets.2024.1301667 (PMC10876796; doi:10.3389/fvets.2024.1301667)
Supplement: Supplementary file 9 [file Data_Sheet_2.PDF]

High clonality of *Mycobacterium avium* subsp. *paratuberculosis* field isolates from red deer revealed by two different methodological approaches of comparative genomic analysis

Silvia Turco[1], Simone Russo[2], Daniele Pietrucci[3], Anita Filippi[2], Marco Milanesi[3], Camilla Luzzago[4], Chiara Garbarino[2], Giorgia Palladini[2], Giovanni Chillemi[3],[5]*, Matteo Ricchi[2]*.
*Corresponding authors: gchillemi@unitus.it, matteo.ricchi@izsler.it

SM1: K10 unmapped CDS retrieved through Reference-based assembly

```
>M01-unmapped_00199 hypothetical protein
MSDPSVCPRCHGQLPAAVGRGRRRLWCSQKCRRAAYEERRAARCGAIGLRVERVVQQVEK
PVRYVEYRDRIVEVTPPPPDPAQAAAIVLTSPRACRVVLDSLTDAVTLGTLHRGEHAATV
RAAARLLTALRAARLLT
>M01-unmapped_00354 TauD/TfdA family dioxygenase MAPK10
MQVAEWGGETEFANSYAAHDALTDDEKDRFGSLRVVHSLEASQRRVDPDPTPEQLARWRS
RRTHEHPLVWTHRTGRKSLVLGASADYVVGMDLDEGRALLAELLDRATLPDKVYSHTWSV
GDTVIWDNRGVLHRAAPYDPSSAREMLRTTVLGDEPIE
>M01-unmapped_00448 HNH endonuclease MAPK10
MEPRPEPEPQREPERAFTRTSTEQGSCYRITLAHDEAAKFDAALQSHLDALIAEWEHDRD
NTERVFDQRPPLPDSLDAFMRLVETGWDAEAARRPHGQHTTVVMHLDIAQRLAALNLGPL
LTDAERQYLTCDATCEVWFEREGQPIGAGRTTRVINRRLRRALEHRHPTCAVPGCGATRG
LHAHHLRHWEDGGPTELAKVRCC
>M01-unmapped_00483 hypothetical protein
MAGEAATAAAAPEAVAGAAIASHISHRQHRDRSPAGASRSGPATNAATQPFTGSPNVTDS
GSEPPAPAAGRSGRVGSSTTPRANSGDPRLVDLGPTGLRSPIDPTSTSPAHQSKGARVKQ
PRQEKGPACVSYRR
>M01-unmapped_00500 hypothetical protein
MRGIDAELIEQSAHRVGEVSERVRLVDAFGRSAVSRHVRHDHAKFLRKRVDVARVVRHAR
GAGSAAVQQQYRRARTLFGEED
>M01-unmapped_00525 hypothetical protein
MHTNITFSTEADIIAAAPAILGFAPTNSIVAYLLHRDTNTGGLQLRSAIRFDVAVSIEQA
ANFPATVNLRPETTHAALLLAVSDEPHEWHALNVLDTASAALRAAGIPVLRRLLTRDVTA
QGQWYDPDSGATGPTYPYTESLVTAHRALSGQRVSTNRSEIEAEFSYLPPAPPMALADHG
ELVIQAAQDIADALEGHPINRSLPTRAGIAITADVAVRNAMIAAAAEHTDTAAYLWTHIG
RRLRGRPRAEALTIAAACYCFQGDRIRAGIAARAALTEAESTQSPPPPLCLMLLTALNSD
VTVEQLGRAIINAVAHP
>M01-unmapped_00538 hypothetical protein
MSTARIDTIASSDRENSKATHSPGAAPCSASRCASRLAAWSSSRYVIDRPSQLIATASGV
CAARAANNTGIDGGAGGGPASAARLPMSSKR
>M01-unmapped_00541 hypothetical protein
MKAIRAAGTAGSIGTYAAPAFSTPRIATTASAERSSSNPTYCPRSTPC
>M01-unmapped_00542 hypothetical protein
MMMQPRRQPMRFQRLTAEHHRLQLQLLTQLRGQRISTLQRIKRRRRLTQHTDLLTNQQRV
QVFW
>M01-unmapped_00555 hypothetical protein
MASFIRAGDQPAVSITRQPMSAWSAGITTWCGVPSQTANTVRRLSCRPTTSSSAAPMASV
SNDPRRCSAMAMLCTGDGPCSWSRNHNRDWANDNGTTAGRSPATTRASVRPPPRTRGANC
ATVGASNNVRTETPAPNTPLTAAINRIADSESPPKSKNESSTPTRPTPNT
>M01-unmapped_00580 hypothetical protein
MIDLAEPAAEGDLGMRIELQVAEDQNSVVFQRIQDGFTNRVVGGQRRRDEAGDLSADRVG
ELGDGEQTHGRSPF
```
