# Supplementary material for: High clonality of Mycobacterium avium subsp. paratuberculosis field isolates from red deer revealed by two different methodological approaches of comparative genomic analysis
Source: Front Vet Sci. 2024 Feb 6;11:1301667. doi: 10.3389/fvets.2024.1301667 (PMC10876796; doi:10.3389/fvets.2024.1301667)
Supplement: Supplementary file 10 [file Data_Sheet_3.PDF]

High clonality of *Mycobacterium avium* subsp. *paratuberculosis* field isolates from red deer revealed by two different methodological approaches of comparative genomic analysis

Silvia Turco[1], Simone Russo[2], Daniele Pietrucci[3], Anita Filippi[2], Marco Milanesi[3], Camilla Luzzago[4], Chiara Garbarino[2], Giorgia Palladini[2], Giovanni Chillemi[3,5]*, Matteo Ricchi[2]*.
*Corresponding authors: gchillemi@unitus.it, matteo.ricchi@izsler.it

SM2: Thirty-three CDS from M04 not present in both M01 and K10

```
>M04-unmapped_00036 hypothetical protein
MNALLALTRTQVWMSAARGFRLSGRTITSDIHRTPPDQDRVRPRHYPPRYCSYLDDNLMNREMYNHVHDDPTRLTSRLAF
RLRRS
>M04-unmapped_00044 hypothetical protein
MSSWDIPVVTALASQRAQLEGSTLRPGPALVMASVVLTTDR
>M04-unmapped_00362 hypothetical protein
MHPNPLVHNGIGADQHNCGITTCSDTQPTATGALTSKQASAGAGHREAFPWP
>M04-unmapped_00411 spirocyclase AveC family protein MAPK10
MFYLVYGLAFASFKWTRIATSVACPWPYPEAKVYDPQGFYEQNGQQGPYSVGIASTWMMAQPHGRPHVTLGSISDRCASN
KQ
>M04-unmapped_00412 hypothetical protein
MSCPAGASGGGALSADGSVITATVLLKGDVGERQKASASLNEHLAKDMPAGINAAMAGNS
PLMGDLTEVDGQDMMTAEMVSLPLAFIVLPLAQRSVLAAMMPMVLGMCAVGVTLGLLSLR
CPSWTGTYSSSRWWR
>M04-unmapped_00413 MMPL family transporter MAPK10
MDWNVFVESLVAMMGLAIGIDYSLLIVRRYREELSAGMVPRQAIVRTLETAGRTALFRA
>M04-unmapped_00414 hypothetical protein
MRRPVPVLVACTLILLVAAIMVAIFRSFCLDRIPEMKQIGFGLAVAVLFDATLIRVTLVP
AFIENHRELELVDPTPPGPAATRTPARMSSRTRFTT
>M04-unmapped_00415 cytochrome P450 MAPK10
MSSETSDLSSGGDMPVMPVPRAVQCPLEPPAAFTTWRQEPGLRRATWQGQPHWVVSRYHD
IREALVDQRLSADTIDWSRMPAGRENTPVMFARTDDPEHHRLRRMMTSQFTFRRSESRRQ
QIQQLVDECLDTMIATGPPSDLVREFALPVPSLVIALLLGVPEEDLDFFQRNTAITLDSS
VSDEQRSQAFAAMYLYIHELTQRKQREPGDDLISRLVTDYVMTGQLDRDTTAMTGVIMMQ
AGHETTANMIALGTLALLDRPEVFHRLGQTDDHSLVANIVEELMRYLTIVQSQVDRVATQ
DLVIGGQLVRAGERLLMNLPAGNWDDTFASDPDQFDVERKTRGHLGFGYGVHQCIGQNLA
RVEMQVAFASLARRLPSLQLAVPSADLTFKAESGIYGTNELPVTW
>M04-unmapped_00416 cytochrome P450 MAPK10
MADIRISEACHGPADTRHYGYEPTFILRGLTNLHLEFTPLD
>M04-unmapped_00496 hypothetical protein
MVGVQRSAPGASAGTTGALGAIGSIRRYGSPCAGRWSFSPLLGHNAAARSAWAVMDSEGL
TPRLAEMAAPSTTYSVG
>M04-unmapped_00533 hypothetical protein
MVAVSVSIAVIALAIARVLSTRARCITSEVCERNSNWPRRGSAVRNTSDAAAVARICAPT
SEPTPWAAAPTASSPAVV
>M04-unmapped_00542 hypothetical protein
MVSASKTSVRNSTVPAMPAGRPSSVQRSHREKVRSIRAVPMSIGSAATSSSPRRSRVGSS
PRQVNIT
>M04-unmapped_00549 SDR family NAD(P)-dependent oxidoreductase MAPK10
MIDVHYVGTVLVTRAAWPHFIRAGYGRVVNTVSEAMLGGIPELSSYGAAKGAVFGLTRNL
ATEGAAHGIAVNAIAPRAFTRMSASHSHALAEHLSIPTDVMDQINAAMPPELCAPAAVFL
AHDSCTLNGEVLQIGMGSVARLAVVRGPGFARDALTAEDIADNLADVMNLHDASVTESAA
PAL
>M04-unmapped_00552 hypothetical protein
MLRSNSDAGVVAVADALLAFFGDAEQIADGAHRHDCTEIGDEVEALGVAQRIQCARTEVT
HQAFYGQHAPWGENA
```

```
>M04-unmapped_00556 hypothetical protein
MSAPRSSRGFARKLAHPVARETNRHVVGHRIGSRAHGDGSVSEDLVITLSYEENVMFTLA
VLLRSSLACEATSVATTAMLAPDSRLLVAAA
>M04-unmapped_00557 hypothetical protein
MRVAWGKSRASRSASEKGNNLSSVPHTTATGLSKRWIASAARRVSAGSNDAVTVAACARV
DLLVRKAPNSSARSPADSRVRHTARRNTF
>M04-unmapped_00559 aldehyde dehydrogenase family protein MAPK10
MSGSLEQSLAVARRLRTGFIGLNGTVGYGADTPFGGFKASGVGRQNGVARFDQSTEVKSV
AYPAS
>M04-unmapped_00565 TetR/AcrR family transcriptional regulator MAPK10
MRDIIEATALGPARVYSVVGSKDRLFRAIIEAFGRKAGGGFAEVLASDATAVEKLDALSW
VNINAIDRFPHEWKIQLAWLRQHPPNTPEPGLAFAQRLAELESLLREGIRSHDLQAHTSS
LEMLARCVMGVLWIPENLVHRLGRRAALRLARDTVIRGIADRSLTPTRGPART
>M04-unmapped_00566 CbbQ/NirQ/NorQ/GpvN family protein MAPK10
MLHPLADHRRQLPVERLGVTLAAAPGFGLVVSYNPGYQSVLKDLKDSTRQRMVALEFDFP
NADIEETIVATEAGISSDHAAQLVRFGQAIRRLETGGLREVASTRVLIAAGRLIAQGLPA
PIAARAAIAGPLSDDPAITNGLIQMIEVYLNENV
>M04-unmapped_00568 hypothetical protein
MKFFTLLEPIYKIVCRMALVVGSEQRPLSPQGLRISLLLDACFTPMLSASP
>M04-unmapped_00571 TNT antitoxin family protein MAPK10
MGVTERYLYGLFGGSVRRNDLPMLKAPFERHELRGGYSIDKQSFAGRERHVLRDRSGSYV
AVAALDRLVTLSHYLGATIEDIKTSFQAPDGKPLFCIWENAQP
>M04-unmapped_00574 hypothetical protein
MGRVARDGSVTVGMPVNERVAGDTRANAVTNVDVLLGPAAIGTDLRAIRAAVGQRQHRQV
PPGPVLGVRPSAGLLG
>M04-unmapped_00578 hypothetical protein
MPAITAPLYSLLGPIFGHIAVILGFGIMHPRVRALVPMPWTRRHDIEFLVFSRVLRGAYR
RLPRRITETPLARNRRQYERIVARYQGVGLVSFVPGATQTCGR
>M04-unmapped_00583 hypothetical protein
MIRWLSARTDCESNRVRTDSAPSTAVLVSAITRIADSESPPSSKKESSIETRSIPSTRAK
MPATVSSVGFVGAMYSPPRFRYSGAGRARVSSLPLTVSGSASSASTADGSM
>M04-unmapped_00586 alpha/beta hydrolase MAPK10
MLLYPSLDLSAETRSRTLFSDGFFLSKQDMDWFKELYLDGAGLAADDARVSPLKAADLSG
LAPALVLTAGFDPLRDEGNEYATALRSAGVSVDHRQFDALTHGFASLAPFGGGSADAIAA
TISAIRAHLSRA
>M04-unmapped_00588 hypothetical protein
MWWMVSSSRCRSSAMRISAARNAGRLARSLTAARSAAHSSSTFCSGSTSSGPSWMYRQAI
TGSAGMTWTGSPYCGQNRAARLGWRLTAFCTASRSRCGSRVPLTVMPSCTA
>M04-unmapped_00589 hypothetical protein
MQEAKDFLIGEFLSVDLGGGQAAGEIVAGLLAAGRKDGHQVVTQRLGGFGGALGIDPY
>M04-unmapped_00593 hypothetical protein
MPPSSPLSSLSEPSELVELTIGGAGSVCGAAATAVPATA
>M04-unmapped_00594 TetR/AcrR family transcriptional regulator MAPK10
MDPSLLVHTCLATVKYVLDLWLTASSLTDPPFHQQLDRALRKALAGF
>M04-unmapped_00598 hypothetical protein
MRPSKVTAGACGLRCACAVNSAGIDVRVAGRSRTARAPQSCSRWCSAGSSTSIDDSGRAG
SAVMATSTRRIRSISVAMSSSSKTRVAYSIWRRSCDPGWACTVSG
>M04-unmapped_00606 hypothetical protein
MPGGNATRVATGPFCGEPPPTRSTGRIDSAIGSAEPDNRFSELVAAPAGLLQYAHVTGDLAVSTRLTCWSG
>M04-unmapped_00607 hypothetical protein
MYSSPTTPSGAGRNHLSSTNSAAPGTGEPIGGAPDSAVSGALIAAYTVASVGP
>M04-unmapped_00608 hypothetical protein
MYSSPTTPGGAGRSHRSSTNSAARGTGDPIGADPVPGCSGVLIAAYTVASVGP
```
